# Effects of debunking interventions on endorsement of alternative medicine: a randomized controlled experiment in Peru

**DOI:** 10.1038/s41598-026-38260-w

**Published:** 2026-02-04

**Authors:** Angelo Fasce, José Rosales-Trabuco, Itxaso Barberia, Elvis Pinedo-Yzaguirre, Juan Manuel Espinoza Nuñez, Wilson Marcos Ortiz-Treviños, Christian David Pizarro-Moncada, Mario Reyes-Bossio, Carlos Carbajal-León, Javier Rodríguez-Ferreiro

**Affiliations:** 1 Institute for Planetary Health Behaviour, Universität Erfurt, Erfurt, Germany; 2 Health Communication Working Group, Bernhard-Nocht-Institut für Tropenmedizin, Hamburg, Germany; 3 Programa de Estudios Generales, Universidad de Lima, Lima, Peru; 4 Grup de Recerca en Cognició i Llenguatge, Departament de Cognició, Desenvolupament i Psicologia de l’Educació, Secció Processos Cognitius, Institut de Neurociències, Universitat de Barcelona, Barcelona, Spain; 5 Departamento Académico de Cursos Básicos, Universidad Científica del Sur, Lima, Peru; 6 Departamento de Humanidades, Universidad Privada del Norte, Trujillo, Peru; 7 Departamento de Educación, Facultad de Educación, Universidad Nacional Mayor de San Marcos, Lima, Peru; 8 Facultad de Psicología, Universidad Peruana de Ciencias Aplicadas, Lima, Peru; 9 Facultad de Ciencias de la Comunicación, Turismo y Psicología, Universidad de San Martín de Porres, Lima, Peru

**Keywords:** Alternative medicine, CAM, Debunking intervention, Health misinformation, Empathetic-refutational interview

## Abstract

Debunking interventions to tackle misconceptions related to scientific issues have gained momentum, especially in the context of health care. In this randomized controlled experiment, we assessed the effectiveness of tailored (i.e., contrarian information addressing participants’ specific motives with an affirmation of their psychological profile) and non-tailored (i.e., general contrarian information regardless of participants’ psychological profile) debunking interventions in a sample of 167 Peruvian participants with positive attitudes toward egg cleanse, a popular local alternative medicine treatment. Our debunking interventions did not significantly correct attitudinal variables related to egg cleanse, such as belief in effectiveness, future use, and preference over conventional medicine. However, exploratory analyses showed significant reductions in endorsement of usage reasons within both the tailored debunk (*d* = 0.50) and non-tailored debunk (*d* = 0.62) groups. Moreover, a comparison between the two debunking groups indicated that participants who received a tailored debunk were more satisfied with the interaction with the physician than those who received a non-tailored debunk (*d* = 0.73). These results suggest that debunking interventions on misinformed health beliefs could have an impact also in the Peruvian cultural context and for alternative medicine, even though more direct attitudinal variables are particularly resistant to change. Strengths and weaknesses of tailored and non-tailored approaches to health misinformation, as well as future research pathways to shed light on the impact and pitfalls of debunking interventions, are discussed.

**Supplementary Information:**

The online version contains supplementary material available at 10.1038/s41598-026-38260-w.

## Introduction

Misinformation around scientific issues has become a recurring and worrying societal issue^1^, especially in the context of health care, as misinformation damages patients’ knowledge, attitudes, and behavioral intentions^2,3^. Nevertheless, tackling health misconceptions is particularly challenging because doing so in the wrong way (i.e., without an adequate level of detail or by attacking the patient’s worldview) can trigger reactance and, potentially, a detrimental backfire effect^4,5^. However, recent large-scale studies indicate that such backfire effects are rare or artifactual, often emerging from measurement error or low reliability rather than genuine reinforcement of false beliefs^6,7^. The current consensus is that corrective interventions generally work as intended, although their magnitude depends on communicative and contextual factors. A range of interventions have been developed to address the psychological drivers of misinformed beliefs, among which debunking interventions represent one of the most extensively researched and empirically supported strategies for countering misinformation and its effects^8^.

Recent work has emphasized the use of debunking interventions in interactions between healthcare professionals and patients, with particular focus on vaccine hesitancy^5^. In this regard, Holford et al.^9^ distinguish between empathetic (i.e., tailored) and non-empathetic (i.e., non-tailored) debunking messages. Unlike general, non-tailored debunking messages, which reflect a communication style that does not attend to the specific motives of the interlocutor, tailored debunking addresses misconceptions by considering the “attitude roots” of the individual’s beliefs—that is, the beliefs, ideologies, fears, and identity issues that motivate people to want to reject a scientific consensus^10,11^. Hence, tailored debunking aligned with the individual’s motivations is based on elicitation of concerns (i.e., open-ended questions and active listening to identify attitude roots), affirmation (i.e., showing empathy toward the patient’s position by expressing understanding of their concerns and motivations), and a tailored debunk (i.e., explaining why the patient’s misconception is wrong without challenging the underlying attitude root)^9^.

With this study, we aim at extending for the first time the assessment of tailored and non-tailored debunking interventions in the health domain to the context of a developing country, Peru, and to misinformation related to *complementary and alternative medicine* (CAM), defined by the World Health Organization^12^ as “a broad set of health care practices that are not part of that country’s own traditional or conventional medicine and are not fully integrated into the dominant health care system” and which "are used interchangeably with traditional medicine in some countries”. Examples of these include homeopathy, crystal healing, reflexology, magnet therapy, and anthroposophic medicine. The use of CAM constitutes a relevant issue for clinical practice due to its associated risks^13,14^ and the recurrent spread of CAM-related misinformation^2,15^. The present experiment focuses on the impact of debunking interventions on endorsement of logically independent usage reasons and positive attitudes toward egg cleanse, a CAM technique widely used in Peru and other parts of Hispanic America^16^. Egg cleanse seeks to purify the spirit and cure illnesses, which can be physical or spiritual (e.g., evil eye). Although egg cleanse presents a wide range of variants, the core practice consists of rubbing a fresh chicken egg, which is deemed a symbol of the creation and development of life, over the patient’s body for the egg to absorb negative energies causing discomfort and illness. At the end of the ritual, the yolk and the white of the egg are poured into a glass and interpreted by the practitioner to diagnose the patient’s spiritual state before the cleanse^17,18^. The Peruvian State does not integrate egg cleanse into the public health system, but other CAM techniques such as phytotherapy, acupuncture, and yoga are offered as part of public health coverage^19^.

In a comprehensive systematic review, Tangkiatkumjai et al.^20^ described several reasons to use CAM, including having an expectation of the health benefit, dissatisfaction with conventional medicine, and perceived safety. These reasons vary across cultural settings—for example, internal locus of control tends to be more common in Western populations, whereas social networks are more salient amongst Asian populations^20^. In the case of Peru, it is important to highlight that it is a pluricultural and multiethnic country, where each ethnic group expresses its culture in diverse ways^21^. In Peru, the dominant Western perspective exists alongside pre-Hispanic worldviews with a differential understanding of the human life cycle, health and illness, and childbirth practices and nurture^22–24^. Additionally, internal migrations have generated complex dynamics between indigenous and non-indigenous populations^25^. These intersections between cultural backgrounds have shaped the syncretic history, culture and sociopolitical landscape of Peru, manifesting itself in practices like egg cleanse, which often incorporates prayers of Christian and Andean origin, and whose presentation to the public varies depending on the region, the ethnicity, and the socioeconomic status of the target population (e.g., using rhetorical styles more prone to pseudoscience or spiritualism). Although practices like egg cleanse are widely used in rural and indigenous sectors of Peruvian society, not only driven by tradition but also by low costs and accessibility^26–29^, these practices are also viewed with skepticism by other sectors of Peruvian society^19,30^.

## Overview of the present experiment

In this randomized controlled experiment, we assessed the effectiveness of two debunking interventions—non-tailored and tailored debunks of usage reasons on a Peruvian sample of participants with positive attitudes toward egg cleanse. In a previous survey, the participants completed a pre-test questionnaire including the following attitudinal variables: belief in the effectiveness of egg cleanse, willingness to use egg cleanse in the future, and preference for egg cleanse over conventional medicine. For the present study, they started rating different usage reasons and selecting their two preferred ones. Then, they were randomly allocated to three groups: a group in which participants received from a fictitious physician a single, general, non-tailored debunk regardless of their preferred usage reasons, a group in which participants received from the same fictitious physician two tailored debunks specifically designed to address their two preferred usage reasons, and a passive control group in which participants received no debunk, thus directly completing the post-test questionnaire (including the questions regarding the attitudinal variables and usage reasons) without experimental manipulation. Both experimental groups completed an additional post-test measure of satisfaction with the interaction with the fictitious physician.

This study was conducted in accordance with the relevant regulations and the Declaration of Helsinki, and received approval from the ethics committee of the Faculty of Health Sciences at the Peruvian University of Applied Sciences (PI 290–24). Informed consent was obtained from all participants.

The experimental design was pre-registered at https://aspredicted.org/zbsp-bhnv.pdf. Although no formal hypotheses were preregistered, our analytic plan and theoretical rationale implied two main expectations^9^. First, both the tailored and the non-tailored debunking interventions were expected to reduce positive attitudes and endorsement of reasons for using the “egg cleanse” compared with the control group. Second, we anticipated higher satisfaction with the refutation among participants receiving tailored debunks compared to participants receiving the non-tailored debunk.

All statistical analyses were conducted using Jamovi (version 2.3.28) for Windows. All tests were two-tailed, and results were considered statistically significant at *p* < 0.05.

## Methods

### Sample

The sample of participants used in this experiment is based on a previous sample collected through NetQuest, a panel provider operating in Peru, to be representative of the general population in terms of age, gender, and region. This previous study constitutes a comprehensive mixed-methods examination of psychosocial predictors, attitudes, and spontaneous usage reasons (using open-ended questions) of CAM in Peru^31^. With the exception of the usage reasons, the pre-test measures of the present experiment were taken from the previous study. For this experiment, after two weeks we recontacted 353 participants who had indicated medium to high levels of belief in the effectiveness of egg cleanse in a previous study (i.e., responses of 4, 5, 6, or 7 on a 1–7 Likert scale). To ensure that the initial level of belief was evenly distributed across the three experimental conditions, participants were randomly assigned to each group using a stratified randomization procedure based on their pretest belief scores. This procedure ensured a balanced representation of participants with scores of 4, 5, 6, and 7 across groups, without requiring exactly equal numbers in each score. A total of 167 participants completed the experiment and passed both attention checks. Of these, 52 were assigned to the tailored debunk group, 56 to the non-tailored debunk group, and 59 to the control group. The sociodemographic characteristics of the sample are displayed in Table S1. Given our sample size, the critical values for detecting group differences at *α* = 0.05 (two-tailed) are approximately *F*(2, 164) = 3.90 and *t* = 1.98 (*α* = 0.05, two-tailed).

### Measures

*Usage reasons* (administered before and after the experimental manipulation): Following a previous psychological classification of anti-vaccination arguments^10,32,33^, used in similar experiments addressing vaccine hesitancy^9^, we developed a psychological classification of common arguments in favor of CAM techniques, such as egg cleanse, based on a prior comprehensive systematic review^20^, which covered preference for a holistic approach to health, preference for natural products and treatments, distrust in conventional medicine, benefits of egg cleanse, reliance on positive testimonies, reliance on personal intuition, ideological traditionalism, and alignment with personal conceptions of spirituality. Participants indicated agreement with each of these usage reasons using a Likert scale of 1 to 7 (1 = Strongly disagree, 7 = Strongly agree). Exploratory factor analyses (EFA) using maximum likelihood estimation and parallel analysis resulted in reliable unidimensional structures in both the pre-test (factor loadings > 0.35, *α* = 0.78) and post-test (factor loadings > 0.42, *α* = 0.84). Both usage reasons assessments included an attention check worded “This is an attention check. Please select answer X”, with X being 3 in one of the questionnaires and 6 in the other.

We also assessed three *attitudinal variables* in relation to egg cleanse as well as other examples of CAM known to be present in Peru (belief in effectiveness, future use, and preference over conventional medicine), using a pre-test obtained from a previous study and a post-test administered after the experimental manipulation. Exploratory factor analyses using maximum likelihood estimation conducted at pre- and post-test stages showed non-convergent solutions and inconsistent communalities, indicating that the items did not load onto a single latent factor. Internal consistency analyses also supported this conclusion, yielding low reliability at pre-test (*α* = 0.55) and moderate reliability at post-test (*α* = 0.73). These results suggest that the three items capture conceptually distinct yet related aspects of attitudes toward egg cleanse, and were therefore analyzed separately rather than combined into a composite score.

1. *Belief in effectiveness*. Participants indicated whether they believed in the effectiveness of egg cleanse for the diagnosis and/or treatment of any disease using a single 1–7 Likert scale (1 = Not effective at all, 7 = Totally effective).
2. *Future use.* Participants indicated whether they would use egg cleanse in the future for the diagnosis and/or treatment of any disease using a single 1–7 Likert scale (1 = Definitely would not use, 7 = Definitely would use).
3. *Preference over medicine*. Participants indicated whether they preferred the use of egg cleanse over the use of conventional medical treatments using a single 1–7 Likert scale (1 = Prefer this technique, 7 = Prefer conventional medicine).

*Satisfaction with the physician* (administered in the two experimental groups after the post-test measures) consisted of five items measuring various aspects of satisfaction with the interaction with the physician: agreement with the debunking information received from the physician, coherence and compellingness of the information received from the physician, perceived competence of the physician, trust in the physician, and openness to continue the conversation. Participants indicated their satisfaction using a Likert scale from 1 to 7. EFA using maximum likelihood estimation resulted in a reliable unidimensional structure, with factor loadings > 0.69 and *α* = 0.89.

The complete wording and descriptive statistics of the questionnaire used in the experiment can be found in tables S2, S3, and S4.

### Procedure

All experimental materials were text-based and presented on-screen within the online survey platform. Participants read the materials at their own pace and proceeded to the next screen once they had finished. The debunking messages followed the same affirmation–refutation structure and were comparable in length (160–190 words) and tone, using consistent empathic language across conditions. To standardize the format across groups, the non-tailored debunk was presented in two separate screens, matching the structure of the tailored condition. This ensures that any differences in outcomes can be attributed to tailoring rather than to wording or presentation differences. The debunking information was delivered in writing in both experimental groups by Dr. Pérez, a female fictitious physician. No picture or other graphical material was used. The physician was introduced in the first person through written text, self-identifying by her surname and professional title (“Hello! I'm Dr. Pérez. I would like us to talk about the reasons why you think egg cleanse is effective”). All affirmations and debunks used in the experiment can be found in Table S4.

After indicating their endorsement of all usage reasons using 1 to 7 Likert scales, participants in the three groups dichotomously selected their two preferred reasons for using egg cleanse (the number of times each usage reason was preferred can be found in Table S5). Participants in the tailored debunk group were exposed to affirmations and debunks specifically designed for their respective two preferred usage reasons. These tailored debunks were developed following the structure of the Empathetic Refutational Interview (ERI)^9,34^. In this framework, refutations are constructed as a collaborative dialogue between communicator and recipient, avoiding explicit appeals to external studies or epistemic authorities^35^. Our materials included only the empathic and reasoning-based stages of the ERI (Steps 1–3; “eliciting concerns”, “affirm”, and “offer a tailored refutation”), without progressing to Step 4 (“provide factual information”), which introduces empirical evidence and appeals to specific scientific publications if prior dialogue fails to promote understanding. The debunks used within the framework of the ERI were co-developed and refined in collaboration with healthcare professionals through the EU Horizon 2020 JITSUVAX project^34,36,37^. This previous experience informed the debunks used in this study to enhance their ecological validity.

For example, a participant who selected benefits and safety of egg cleanse and ideological traditionalism as their two most supported reasons would have first been exposed to a screen displaying a tailored debunk for benefits and safety of egg cleanse, comprised of the following affirmation and refutational message:

*“It is normal to have questions and doubts about medical treatments and how they can affect us. We would all like medical treatments to be effective for everyone and in all conditions, but they cannot be guaranteed, like any other product, to be 100% safe and effective. Sometimes it is difficult to face uncertainty, so fear and rejection are perfectly understandable. Many people turn to alternative and traditional medicine without negative consequences, and many of these alternative remedies are presented as safer and more natural than conventional medical treatments.*

*The potential lack of side effects of egg cleanse is not because it is a better treatment, but because it has not been proven to actually have a real therapeutic effect on our body. Since a medical intervention, with a real effect on our body, can always have side effects, unlike egg cleanse, medical treatments undergo strict safety controls. Even though approved medical treatments are not 100% effective, they are approved because their benefits far outweigh any potential adverse effects. Moreover, if we wait to be absolutely certain of safety, we would never do anything in life. Imagine if we refused to get into a car unless the driver could prove 100% that we would not have an accident. Public health institutions and independent researchers have very reliable monitoring systems in place to track all possible side effects of drugs and other medical interventions, using statistics and taking into account many potential causes. However, we are often unaware of the effects and how responsibly egg cleanse is applied.”*

Subsequently, the participant would have been exposed to a second screen displaying the following affirmation and refutational message for ideological traditionalism:

*“Many of our traditions shape and give meaning to the way we act and identify ourselves, so they are part of who we are. People have the right to have their traditions taken into account by health professionals and to be treated with respect regardless of their cultural origin. In general, it is a positive thing to have appreciation for one’s own culture, since it helps to preserve and develop it.*

*Practices that can be considered traditional, such as egg cleanse, do not have evidence of effectiveness when tested using rigorous scientific methods. Therefore, the use of egg cleanse, although it may seem old, can prevent us from receiving truly effective treatment, or deviate us from a healthy lifestyle by confusing the real origin of diseases. Having a lifestyle based on cultural traditions does not in any way imply endangering our lives and those of others, especially those who belong to risk groups or who have other traditions. Furthermore, just because egg cleanse is presented as something ancient or traditional does not mean that it is good for us or our societies to continue practicing it. Traditions are not sacred, unchangeable, immutable or good in themselves; they are constantly changing to suit our needs and ethical principles. Think of all the traditions that have changed throughout our lifetime in favor of healthier practices (for example, smoking in front of children or driving without a seat belt). We must keep ourselves alive if we want, in turn, to keep our traditions alive.”*

In contrast, all participants assigned to the non-tailored debunk group received the same debunk, with no affirmation, regardless of their preferred usage reasons. This general, non-tailored debunk was based on general issues such as the lack of scientific evidence supporting the clinical effectiveness of egg cleanse, the lack of professional regulation and quality control of practitioners of egg cleanse, or the possibility that alternative health beliefs related to egg cleanse negatively affect our decision making on healthy lifestyle and therapeutic opportunities:

*“Alternative medicine techniques such as egg cleanse have not been proven effective for treating diseases and therefore should not be used as a treatment. A lot of effort has been put into studying these types of practices and their effectiveness has never been conclusively proven, using the best tools available for scientific research. Because of this, doctors should not recommend their use, instead recommending treatments that have been proven effective. In addition, people may confuse the real origin of their disease, which can lead to unhealthy lifestyles, as well as losing the opportunity to receive effective treatment to treat their disease. Another thing that can happen is that the person administering egg cleanse, who is usually not a registered and controlled doctor, commits some negligent practice that causes additional damage to the disease itself. Because of all this, I do not recommend using egg cleanse to treat diseases.*

*The use of alternative medicine techniques such as egg cleanse is not recommended by medical associations and scientifically endorsed treatment guides, since we do not have evidence to support its use. On the other hand, the potential dangers of techniques such as egg cleanse are well documented, since they can generate distorted ideas regarding the origin and treatment of diseases, as well as cause patients not to use treatments that are endorsed. Medicine is a science that develops at great speed and that has very powerful tools to generate knowledge and develop treatments that work. Alternative medicine such as egg cleanse, on the other hand, does not have evidence and, consequently, its use for the treatment of diseases is not recommended from a medical point of view.”*

## Results

### Differences in attitudes toward egg cleanse

A series of preregistered analyses of variance were conducted to assess differences between groups in the amount of change. Each of these analyses consisted of a one-way between-subjects analysis of variance (ANOVA) comparing the three experimental conditions (tailored, non-tailored, and control) on change scores (post-test minus pre-test) in the three single-item variables expressing attitudes toward egg cleanse, resulting in non-significant results: belief in effectiveness [*F*(2, 164) = 2.20, *p* > 0.05], future use [*F*(2, 164) = 0.87, *p* > 0.05], and preference over medicine [*F*(2, 164) = 0.93, *p* > 0.05]. The lack of significant effects regarding belief in effectiveness extended to other traditional or alternative disciplines assessed before and after the intervention [*F*(2, 164) = 2.138, *p* > 0.05].

As a robustness check, we additionally conducted non-preregistered analyses of covariance (ANCOVAs), entering the corresponding pre-test scores as covariates (Table S6). When controlling for baseline scores, small but statistically significant effects of experimental condition emerged for belief in the effectiveness of egg cleanse and for perceived effectiveness of other traditional or alternative techniques. Given that these ANCOVAs were conducted as supplementary robustness checks and yielded small effect sizes, these results should be interpreted with caution.

### Differences in satisfaction with the physician

**Fig. 1:**
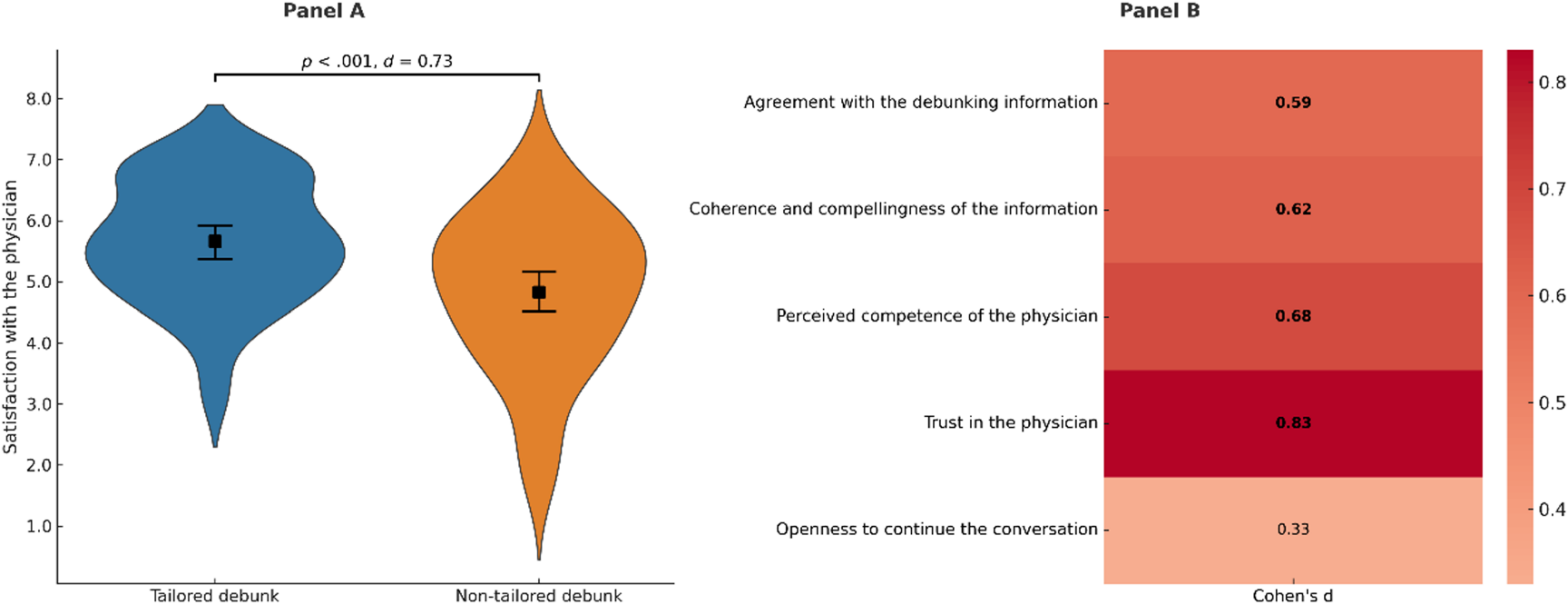
Effect of debunking interventions on satisfaction with the physician. Panel (**A**). Mean satisfaction with the physician across experimental conditions. The shape markers indicate group means with 95% confidence intervals. The difference between experimental groups is displayed with the corresponding *p* and Cohen’s *d* values. Panel (**B**). Effect sizes (Cohen’s *d*) for differences between experimental groups in specific dimensions of satisfaction with the physician. Bolded values indicate statistically significant differences (*p* < .05). All significant results remained after the Bonferroni correction (*p* < .01). Color intensity reflects the magnitude of the effect size.

Preregistered mean comparisons of the responses to the satisfaction questions between the two experimental groups indicated significantly higher satisfaction scores for the tailored group in four of the questions: agreement [*t*(106) = 3.08, *p* < 0.001], coherence [*t*(106) = 3.22, *p* < 0.01], competence [*t*(106) = 3.54, *p* < 0.001], and trust [*t*(106) = 4.32, *p* < 0.001]. These results are summarized in Fig. 1, showing averaged scores for the five satisfaction questions (Panel A) and effect sizes for the comparisons of the responses to each individual question (Panel B). All significant results remained after the Bonferroni correction (*p* < 0.01).

Preregistered correlation analyses between responses to the satisfaction questions and belief change showed a positive association only with regard to the perceived coherence of the physician (*r* = 0.20, *p* < 0.05).

### Differences in usage reasons for egg cleanse

**Fig. 2:**
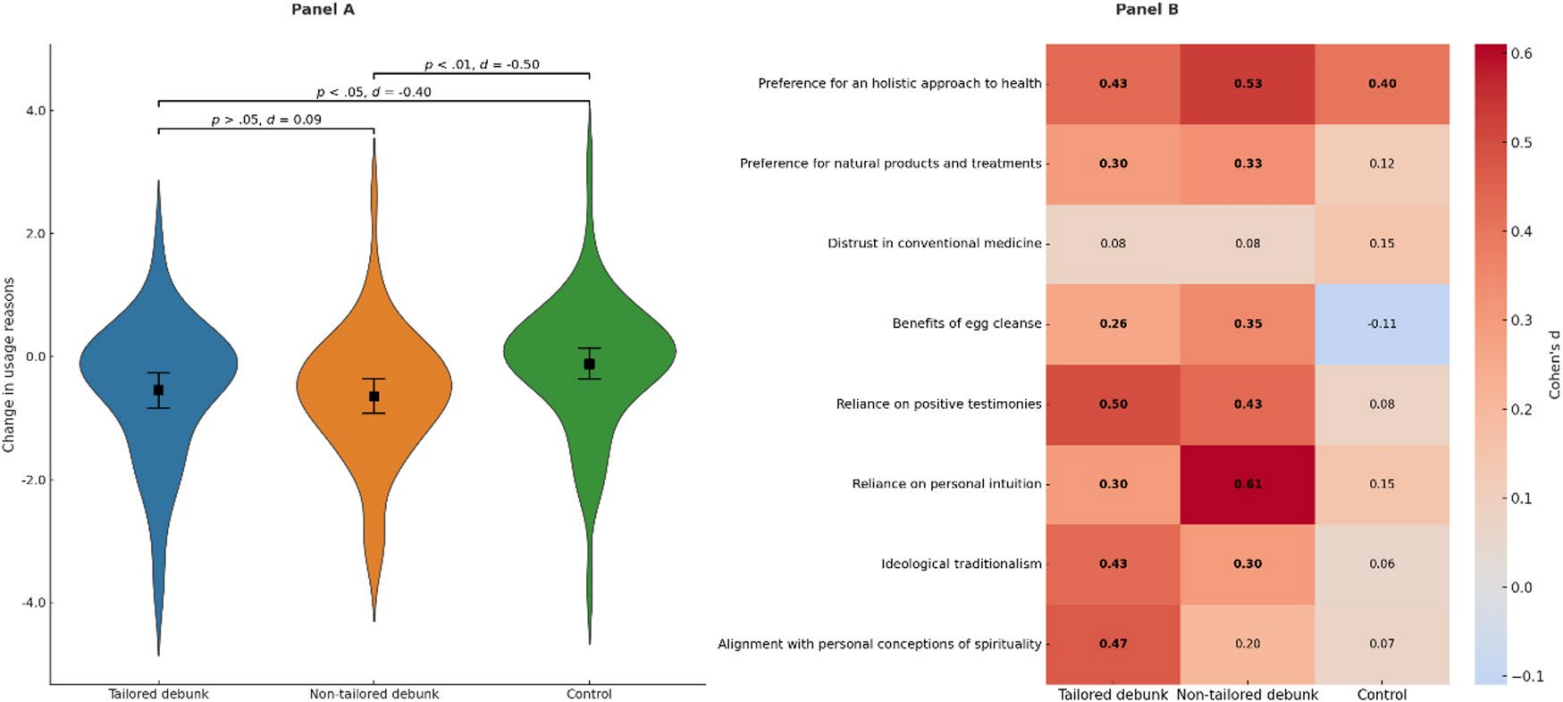
Effect of debunking interventions on usage reasons. Panel (**A**). Mean change in endorsement of usage reasons across experimental conditions. The shape markers indicate group means with 95% confidence intervals. Differences between groups are displayed with the corresponding *p* and Cohen’s *d* values. Panel (**B**). Effect sizes (Cohen’s d) for pre-post changes in endorsement of specific usage reasons across experimental conditions. Values represent Cohen’s d for the difference between pre- and post-intervention ratings within each group. Bolded values indicate statistically significant changes (*p* < .05). Given the large number of multiple comparisons, only two effects remained significant after the Bonferroni corrections (*p* < .002): reliance on positive testimonies within the tailored debunk group, and preference for a holistic approach to health within the non-tailored debunk group. Color intensity reflects the magnitude of the effect size.

We further explored the results by running non-preregistered analyses of the impact of the intervention over usage reasons. This one-way between-subjects ANOVA used the change in mean scores (post-test minus pre-test) as the dependent variable to compare the three experimental conditions (tailored, non-tailored, and control). Change in global scores (i.e., the combined mean of the 8-item scale) is represented in Fig. 2’s Panel A. The ANOVA showed differences between groups in the amount of change in usage reasons [*F*(2, 164) = 3.97, *p* < 0.05]. As a robustness check, a non-preregistered ANCOVA controlling for pre-test usage reasons yielded results consistent with the main ANOVA (Table S6).

Subsequent pairwise comparisons indicated that each of the debunk groups significantly differed from the control group [tailored debunk: *t*(109) = 2.09, *p* < 0.05, *d* = 0.40; non-tailored debunk: *t*(113) = 2.69, *p* < 0.01, *d* = 0.50]. Within groups analyses indicated significant decreases in endorsement of usage reasons in the tailored debunk group [pre-test *M*(*SD*) = 5.10(0.93), post-test *M*(*SD*) = 4.56(1.16); *t*(51) = 3.61, *p* < 0.001; *d* = 0.50] and the non-tailored debunk group [pre-test *M*(*SD*) = 4.90(0.90), post-test *M*(*SD*) = 4.25(1.16); *t*(55) = 4.67, *p* < 0.001; *d* = 0.62]; no significant differences were observed in the control group [pre-test *M*(*SD*) = 4.88(1.04), post-test *M*(*SD*) = 4.76(1.14); *t*(58) = 0.90, *p* > 0.05]. A summary of the analyses at the item level can be found in Fig. 2’s Panel B. Given the large number of multiple comparisons, Bonferroni corrections were applied (adjusted significance threshold *p* < 0.002). Only two effects remained significant after correction: reliance on positive testimonies within the tailored debunk group, and preference for a holistic approach to health within the non-tailored debunk group.

Finally, correlation analyses indicated positive associations between mean change in usage reasons and responses to the agreement (*r* = 0.26, *p* < 0.01) and competence (*r* = 0.19, *p* < 0.05) satisfaction questions.

## Discussion

The reported randomized controlled experiment on the impact of tailored and non-tailored debunking interventions on egg cleanse, a CAM technique popular in Peru, resulted in significant reductions in endorsement of usage reasons in both debunking groups. However, the results suggest greater difficulties for debunking interventions in achieving a reduction in the three key attitudinal variables assessed in the experiment (i.e., belief in the effectiveness of egg cleanse, willingness to use egg cleanse in the future, and preference for egg cleanse over conventional medicine). These difficulties in reducing positive attitudes towards health misconceptions are in line with what has been observed in prior experiments on vaccine hesitancy. Holford et al.^9^ found that debunking interventions did not cause a substantial reduction in psychological antecedents of vaccination^38^, despite participants positively evaluating tailored debunks. Similarly, Schmid and Betsch^5^ observed that a prebunking intervention on mRNA vaccination misinformation did not affect vaccination intentions despite reducing belief in misinformation. This suggests that behavioral intentions and internalized personal preferences are more resistant to change than misinformed beliefs (in this study, the usage reasons), especially in online experiments with a single, rapid intervention.

The present experiment should be regarded as a preliminary step toward developing and empirically refining tailored debunking approaches for health-related misinformation. While the interventions reduced endorsement of usage reasons, identifying more effective formats for attitudinal change remains an important direction for future research. In this regard, research extending the assessment of debunking interventions in two directions is needed: field research in real medical consultations and experimental designs with repeated interventions. A crucial step in this direction would be to adapt to the context of CAM training programs for health care professionals on tailored and non-tailored refutational interviews to address health misinformation^37^, considering the proclivity among some health care professionals to endorse CAM themselves^39^.

Face-to-face interactions with health care professionals might lead to a deeper attitudinal change, which could occur over time after being repeatedly exposed to contrarian information. In this regard, the use of tailored debunks seems to be especially promising in the medium and long term, given the greater satisfaction with the interaction with the physician observed in this and previous experiments^9^. Patient satisfaction, when raised by a tailored communication style, as well as the suitability of empathy for addressing usage reasons prone to reactance^4,5^, could boost patients’ openness to establishing long-term communication with physicians about CAM. The higher satisfaction observed in the tailored group is consistent with the structure of the ERI, in which the affirmation phase precedes the refutation. This initial empathetic exchange may enhance participants’ perception of the communicator’s warmth and competence, contributing to greater satisfaction without necessarily implying higher persuasive impact. Future research could experimentally disentangle the specific contribution of affirmation within the ERI framework to better understand its role in effective debunking. Nevertheless, our results also suggest that non-tailored debunks could constitute a useful tool, being especially promising for mass and short-term interventions. Non-tailored debunks, which usually combine debunking information about several attitude roots in a single message, tend to be less time-intensive, and require less training and the deployment of fewer communication skills. In addition, video-based formats could provide a scalable and low-cost means of delivering these interventions to wider audiences, potentially bridging the gap between face-to-face empathy and large-scale communication.

The reported experiment benefits from multiple strengths such as a randomized and controlled experimental design, a sample of participants with positive attitudes toward CAM from a relevant socio-cultural context given both Peru’s high CAM usage rate and underrepresentation in the current scientific literature, and comprehensive assessment of variables with proper psychometric validity. However, we would like to remark on some limitations. First, although the sample was adequately powered for the reported analyses, its size limits the generalizability of the findings. Accordingly, the conclusions should be interpreted with caution and replicated using larger samples. Second, the debunking messages did not include Step 4 of the ERI, which explicitly presents empirical evidence after the empathetic dialogue. Future studies could incorporate this component to examine whether the addition of direct scientific evidence increases the perceived credibility and persuasive impact of the intervention. Third, although the list of usage reasons assessed in this experiment was adapted from a previous systematic review and empirically validated in the same Peruvian sample^20,31^, it might not be sufficiently comprehensive or detailed to capture all attitude roots underlying CAM use in Peru. Some relevant motivational domains previously identified in Peru—such as paranormal or spiritual beliefs—were not represented among the usage reasons included in the experiment^31^. This omission was mainly due to the practical constraints of recontacting participants within a short time frame and the technical complexity of the natural language processing (NLP) analyses required to extract such categories from open-ended responses, which involved multiple steps including chunking, labeling, and visualization. The assessment of tailored debunks would benefit from future endeavors to construct a more exhaustive and cross-cultural taxonomy of attitude roots motivating CAM use, analogous to the work already done on vaccine hesitancy using systematic review, surveys, and natural language processing^10,32,33^. Fourth, future studies could complement experimental group comparisons with regression or mediation analyses to explore the specific psychological factors and individual differences that may modulate the effectiveness of tailored versus non-tailored debunking interventions. It would also be of great value for future research to focus on specific profiles of CAM users, given that previous research outcomes suggest that the effect of tailored debunks is larger in subsamples with worse attitudes toward vaccination^9^. Similarly, it is possible for tailored debunks of CAM usage reasons to be particularly effective at managing reactance and self-defensive cognition among people strongly committed to CAM.

## Concluding remarks

Tailored and non-tailored debunking interventions against egg cleanse did not significantly impact attitudinal variables such as belief in its effectiveness, future use intentions, and preference over conventional medicine. However, these interventions were shown to be effective in reducing endorsement of common usage reasons for egg cleanse, a popular CAM technique in Peru. Moreover, tailored debunks generated increased satisfaction with the interaction with the physician. More research is needed to further understand the impact and pitfalls of the implementation of debunking interventions to address health misinformation, especially in developing countries where CAM usage rates are particularly high.

## Supplementary Information

Below is the link to the electronic supplementary material.

Supplementary Material 1

## Data Availability

All data and codes used in the study are available at https://www.osf.io/9xbzq.

## References

[1] Lewandowsky, S. et al. When science becomes embroiled in conflict: recognizing the public’s need for debate while combating conspiracies and misinformation. *Ann. Am. Acad. Polit. Soc. Sci.* **700**, 26–40. 10.1177/00027162221084663 (2022). 10.1177/00027162221084663 PMC7613792 36338265

[2] Ernst, E. *Alternative medicine: a critical assessment of 202 modalities* (Copernicus Books, 2022).

[3] Schmid, P., Altay, S. & Scherer, L. The psychological impacts and message features of health misinformation. *Eur. Psychol.* **28**, 162–172. 10.1027/1016-9040/a000494 (2023).

[4] Karlsson, L. et al. Testing psychological inoculation to reduce reactance to vaccine-related communication. *Health Commun.* **39**, 3450–3458. 10.1080/10410236.2024.2325185 (2024). 38450609 10.1080/10410236.2024.2325185

[5] Schmid, P. & Betsch, C. Benefits and pitfalls of debunking interventions to counter mRNA vaccination misinformation during the COVID-19 pandemic. *Sci. Commun.* **44**, 531–558. 10.1177/10755470221129608 (2022). 38603361 10.1177/10755470221129608 PMC9574536

[6] Swire-Thompson, B., DeGutis, J. & Lazer, D. Searching for the backfire effect: measurement and design considerations. *J. Appl. Res. Mem. Cogn.* **9**, 286–299. 10.1016/j.jarmac.2020.06.006 (2020). 32905023 10.1016/j.jarmac.2020.06.006 PMC7462781

[7] Swire-Thompson, B., Miklaucic, N., Wihbey, J. P., Lazer, D. & DeGutis, J. The backfire effect after correcting misinformation is strongly associated with reliability. *J. Exp. Psychol. Gen.* **151**, 1655–1665. 10.1037/xge0001131 (2022). 35130012 10.1037/xge0001131 PMC9283209

[8] Ecker, U. et al. The psychological drivers of misinformation belief and its resistance to correction. *Nat. Rev. Psychol.* **1**, 13–29. 10.1038/s44159-021-00006-y (2022).

[9] Holford, D. L., Schmid, P., Fasce, A. & Lewandowsky, S. The empathetic refutational interview to tackle vaccine misconceptions: four randomized experiments. *Health Psychol.* **43**, 426–437. 10.1037/hea0001354 (2024). 38436659 10.1037/hea0001354

[10] Fasce, A. et al. A taxonomy of anti-vaccination arguments from a systematic literature review and text modelling. *Nat. Hum. Behav.* **7**, 1462–1480. 10.1038/s41562-023-01644-3 (2023). 37460761 10.1038/s41562-023-01644-3

[11] Hornsey, M. J. Why facts are not enough: understanding and managing the motivated rejection of science. *Curr. Direct. Psychol. Sci.* **29**, 583–591. 10.1177/0963721420969364 (2020).

[12] World Health Organization. *Who Global Report on Traditional and Complementary Medicine 2019*. https://apps.who.int/iris/handle/10665/312342 (2019).

[13] Niggemann, B. & Grüber, C. Side-effects of complementary and alternative medicine. *Allergy* **58**, 707–716. 10.1034/j.1398-9995.2003.00219.x (2023). 10.1034/j.1398-9995.2003.00219.x 12859546

[14] Wardle, J. L. & Adams, J. Indirect and non-health risks associated with complementary and alternative medicine use: An integrative review. *Eur. J. Integr. Med.* **6**, 409–422. 10.1016/j.eujim.2014.01.001 (2014).

[15] Recuero, R., Soares, F. & Zago, G. Polarization, hyperpartisanship and echo chambers: How the disinformation about COVID-19 circulates on Twitter. *Contracampo.*10.22409/contracampo.v40i1.45611 (2021).

[16] Mejía Gálvez, J. A., Carrasco, E., Miguel, J. L. & Flores, S. A. Conocimiento, aceptación y uso de medicina tradicional peruana y de medicina alternativa/complementaria en usuarios de consulta externa en Lima Metropolitana. *Revista Peruana de Medicina Integrativa.* **2**, 47–57. 10.26722/rpmi.2017.21.44 (2017).

[17] Nexos. *Botando la cáscara negativa: la limpia de huevo* https://nexos.ulima.edu.pe/2023/11/30/botando-la-cascara-negativa-la-limpia-de-huevo/ (2023).

[18] La República. *¿Por qué razón se usa el huevo en las limpias contra las malas energías?* https://larepublica.pe/datos-lr/respuestas/2022/02/18/por-que-utilizan-el-huevo-como-un-ritual-para-eliminar-las-energias-negativas-evat (2022).

[19] García, R. Medicina tradicional o complementaria: Pacientes que lo usan al mismo tiempo que su tratamiento farmacológico. *Ciencia y Desarrollo.* **22**, 25–30. 10.21503/cyd.v22i1.1735 (2019).

[20] Tangkiatkumjai, M., Boardman, H. & Walker, D. M. Potential factors that influence usage of complementary and alternative medicine worldwide: A systematic review. *BMC Complement. Med. Ther.* **20**, 363. 10.1186/s12906-020-03157-2 (2020). 33228697 10.1186/s12906-020-03157-2 PMC7686746

[21] Campos Briceño, G. R., & Condor Iturrizaga, R. M. La etnicidad en el Perú y su naturaleza multidimensional: una propuesta de medición. *Desde el Sur.* **14**, 1–24 (2022). 10.21142/des-1401-2022-0012

[22] Santa María, L.A. Intercultural health: The life cycle stages in the Andes. *Revista Peruana de Medicina Experimental y Salud Publica.* **34**, 293–298 (2017). 10.17843/rpmesp.2017.342.2732 10.17843/rpmesp.2017.342.2732 29177391

[23] Flores Rojas, M. X. VIH/sida awajún: nociones y experiencias de enfermedad y daño en un contexto de epidemia en la Amazonía Peruana. *Anthropologica* **38**, 235–266. 10.18800/anthropologica.202001.010 (2020).

[24] Del Mastro, I. et al. Home birth preference, childbirth, and newborn care practices in rural Peruvian Amazon. *PLoS ONE* **16**, e0250702. 10.1371/journal.pone.0250702 (2021). 33945560 10.1371/journal.pone.0250702 PMC8096074

[25] Barrón, M. Exclusion and discrimination as sources of inter-ethnic inequality in Peru. *Economia* **31**, 51–80. 10.18800/economia.200801.003 (2008).

[26] Córdoba-Villota, E. E. & Velásquez-Mantilla, D. A. Ancestral knowledge the midwives of traditional medicine, immemorial learnings that are still preserved. *Techno Rev. Int. Technol. Sci. Soc. Rev.* **13**, 1–10 (2023). 10.37467/revtechno.v13.4797

[27] León, G. B., Acosta, M., Saavedra, M. E. & Almonacid, S. Traditional medicine as a treatment for COVID-19 in students and family members at a university in the mountains of Peru. *Prim. Care* **55**, 102526. 10.1016/j.aprim.2022.102526 (2023). 10.1016/j.aprim.2022.102526 PMC9659508 36473428

[28] Santiváñez-Acosta, R., Valenzuela-Oré, F. & Angulo-Bazán, Y. Use of complementary and alternative medicine therapies in the Coronel Portillo province, Ucayali, Peru. *Rev. Peru. Med. Exp. Salud Publica* **37**, 510–515 (2020). 33295554 10.17843/rpmesp.2020.373.4939

[29] Villena-Tejada, M. et al. Use of medicinal plants for COVID-19 prevention and respiratory symptom treatment during the pandemic in Cusco, Peru: A cross-sectional survey. *PLoS ONE* **16**, e0257165. 10.1371/journal.pone.0257165 (2021). 34550994 10.1371/journal.pone.0257165 PMC8457479

[30] Luján-Carpio, E. et al. El servicio de medicina complementaria de EsSalud, una alternativa en el sistema de salud peruano. *Revista Médica Herediana*. **25**, 105–106 (2014). 10.20453/rmh.v25i2.255

[31] Fasce, A. et al. Psychosocial predictors and justification patterns of traditional and alternative medicine in Peru (2025). Preprint at 10.31219/osf.io/7kg3v

[32] Holford, D. L., Fasce, A., Costello, T. & Lewandowsky, S. Psychological profiles of anti-vaccination argument endorsement. *Sci. Rep.* **13**, 11219. 10.1038/s41598-023-30883-7 (2023). 37460585 10.1038/s41598-023-30883-7 PMC10352341

[33] Holford, D. L., Lopez-Lopez, E., Fasce, A., Karlsson, L. & Lewandowsky, S. Identifying the underlying psychological constructs from self-expressed anti-vaccination argumentation. *Hum. Soc. Sci. Commun.* **11**, 926. 10.1057/s41599-024-03416-4 (2024).

[34] Fasce, A. et al. A field test of empathetic refutational and motivational interviewing to address vaccine hesitancy among patients. *npj Vaccines.* **10**, 142 (2025). 10.1038/s41541-025-01197-8 10.1038/s41541-025-01197-8 PMC12229542 40610488

[35] JITSUVAX. *Vaccine attitudes resource* https://jitsuvax.info/welcome/ (2025).

[36] Holford, D. et al. Implementing psychology-based empathetic refutational interview training to support vaccine-confident conversations for health workers. *Preprint at*10.1101/2025.10.08.25337588 (2025). 10.1016/j.puhe.2026.106295 42191453

[37] Holford, D. L. et al. A randomized controlled trial of empathetic refutational learning with health care professionals. *BMC Public Health* **25**, 583. 10.1186/s12889-025-21787-4 (2025). 39939961 10.1186/s12889-025-21787-4 PMC11823235

[38] Betsch, C. et al. Beyond confidence: Development of a measure assessing the 5C psychological antecedents of vaccination. *PLoS ONE* **13**, e0208601. 10.1371/journal.pone.0208601 (2018). 30532274 10.1371/journal.pone.0208601 PMC6285469

[39] Fasce, A. et al. Endorsement of alternative medicine and vaccine hesitancy among physicians: a cross-sectional study in four European countries. *Hum. Vacc. Immunother.* **19**, 2242748. 10.1080/21645515.2023.2242748 (2023). 10.1080/21645515.2023.2242748 PMC10431744 37581343

