# Supplementary material for: Effects of debunking interventions on endorsement of alternative medicine: a randomized controlled experiment in Peru
Source: Sci Rep. 2026 Feb 4;16:4995. doi: 10.1038/s41598-026-38260-w (PMC12876895; doi:10.1038/s41598-026-38260-w)
Supplement: Supplementary file 1 — Supplementary Material 1 [file 41598_2026_38260_MOESM1_ESM.pdf]

Supplementary Material for *Debunking interventions on endorsement of alternative medicine: a randomized controlled experiment in Peru*

Angelo Fasce[1,2], José Rosales-Trabuco[3], Itxaso Barberia[4], Elvis Pinedo-Yzaguirre[5], Juan Manuel Espinoza Nuñez[6], Wilson Marcos Ortiz-Treviños[7], Christian David Pizarro Moncada[6], Mario Reyes-Bossio[8], Carlos Carbajal-León[9], and Javier Rodríguez-Ferreiro[4]

1. Institute for Planetary Health Behaviour, Universität Erfurt, Germany
2. Health Communication Working Group, Bernhard-Nocht-Institut für Tropenmedizin, Germany
3. Programa de Estudios Generales, Universidad de Lima, Peru
4. Grup de Recerca en Cognició i Llenguatge, Departament de Cognició, Desenvolupament i Psicologia de l'Educació, Secció Processos Cognitius, Institut de Neurociències, Universitat de Barcelona, Spain
5. Departamento Académico de Cursos Básicos, Universidad Científica del Sur, Peru
6. Departamento de Humanidades, Universidad Privada del Norte, Peru
7. Facultad de Educación, Departamento de Educación, Universidad Nacional Mayor de San Marcos, Peru
8. Facultad de Psicología, Universidad Peruana de Ciencias Aplicadas, Peru
9. Facultad de Ciencias de la Comunicación, Turismo y Psicología, Universidad de San Martín de Porres, Peru

Table S1

Sociodemographic characteristics of the sample.

Variable	Response option	Percentage	M(SD)
Age			41.15(14.72)
	18-28	25.7%	
	29-39	24%	
	40-50	22.2%	
	51-61	17.3%	
	62-72	10.8%	
Gender			
	Male	44.9%	
	Female	51.1%	
Region			
	Lima	56.9%	
	South	17.4%	
	Center	6%	
	North	19.8%	
Religiosity			3.12(0.56)
	Atheist	1.2%	
	Agnostic	6.6%	
	Non-practicing religious	71.3%	
	Practicing religious	21%	
Education			3.80(1.32)
	Primary	1.2%	
	Secondary	19.8%	
	Technical	23.4%	
	Undergraduate	18%	
	Graduate	29.3%	
	Postgraduate	8.4%	
Political Orientation			4.60(1.38)
	Far left	3%	
	2	1.2%	

3	7.2%
4	47.3%
5	19.2%
6	6.6%
Far right	15.6%

Note: All data in this table are descriptive (percentages, means, and standard deviations). No inferential tests were performed.

Table S2

Items on attitudes toward egg cleanse and satisfaction with the interaction with the physician.

Type of variable	Concept	Question	Response options
Attitudes toward egg cleanse			
	Belief in effectiveness	Do you believe in the effectiveness of egg cleanse for the diagnosis and/or treatment of any disease?	1= Not effective at all, 7 =Totally effective
	Future use	Would you use egg cleanse in the future for the diagnosis and/or treatment of any disease?	1 = Definitely would not use, 7 = Definitely would use
	Preference over medicine	Do you prefer the use of egg cleanse to the use of conventional medical treatments?	1 = Prefer this technique, 7 = Prefer conventional medicine
Satisfaction with the physician			
	Agreement	Do you agree with the information that Dr. Pérez has given you?	1 = Strongly disagree, 7 = Strongly agree
	Satisfaction	Regardless of whether you agree with it, do you find the information coherent and convincing?	1 = Not at all, 7 = Totally
	Competence	How competent do you find Dr. Pérez?	1 = Not competent at all, 7 = Totally competent
	Trust	Do you trust Dr. Pérez?	1 =Not at all, 7 = Totally
	Openness	How open are you to further conversations with Dr. Pérez?	1 = Not at all, 7 = Totally

Table S3

Descriptive statistics of usage reasons, attitudinal variables and satisfaction.

Variable	Item	Response option	Percentage	M(SD)
Pre-test usage reasons				4.95(0.96)
	Pre-test holistic approach			4.94(1.46)
		1	3%	
		2	3%	
		3	4.2%	
		4	31.7%	
		5	20.4%	
		6	20.4%	
		7	17.4%	
	Pre-test natural treatments			5.18(1.50)
		1	3.6%	
		2	1.8%	
		3	3%	
		4	24.6%	
		5	23.4%	
		6	19.2%	
		7	24.6%	
	Pre-test dissatisfaction			4.43(1.81)
		1	10.2%	
		2	6.6%	
		3	4.8%	
		4	34.7%	
		5	13.8%	
		6	12%	
		7	18%	
	Pre-test benefits and security			4.56(1.47)
		1	5.4%	
		2	4.2%	

		3	4.8%	
		4	36.5%	
		5	22.8%	
		6	16.8%	
		7	9.6%	
	Pre-test personal testimonies			5.40(1.43)
		1	3%	
		2	1.2%	
		3	1.8%	
		4	21%	
		5	19.8%	
		6	26.9%	
		7	26.3%	
	Pre-test personal intuition			4.88(1.47)
		1	4.2%	
		2	1.2%	
		3	6%	
		4	29.9%	
		5	25.7%	
		6	15.6%	
		7	17.4%	
	Pre-test traditionalism			5.24(1.53)
		1	3.6%	
		2	1.8%	
		3	4.2%	
		4	21.6%	
		5	21%	
		6	22.2%	
		7	25.7%	
	Pre-test spirituality			5.01(1.49)
		1	3%	
		2	3.6%	
		3	4.8%	
		4	25.1%	
		5	24%	
		6	20.4%	
		7	19.2%	
Pre-test attitudes toward egg cleanse				
	Pre-test belief in effectiveness			5.37(1.13)
		1	-	
		2	-	
		3	-	
		4	29.3%	
		5	27.5%	
		6	20.4%	
		7	22.8%	
	Pre-test future use			4.89(1.76)
		1	4.2%	
		2	7.2%	
		3	9%	
		4	21.6%	
		5	16.8%	
		6	15.6%	
		7	25.7%	
	Pre-test preference over medicine			4.01(1.98)
		1	17.4%	
		2	7.8%	
		3	10.2%	
		4	26.3%	
		5	13.2%	
		6	9.6%	
		7	15.6%	
Post-test usage reasons				4.52(1.16)
	Post-test holistic approach			4.19(1.61)
		1	9.6%	
		2	5.4%	
		3	9.6%	
		4	35.9%	
		5	19.2%	
		6	12%	
		7	8.4%	
	Post-test natural treatments			4.75(1.66)
		1	7.2%	
		2	4.2%	
		3	3%	
		4	29.3%	

		5	21.6%	
		6	18%	
		7	16.8%	
	Post-test dissatisfaction			4.25(1.96)
		1	13.2%	
		2	9.6%	
		3	5.4%	
		4	31.1%	
		5	12%	
		6	9%	
		7	19.8%	
	Post-test benefits and security			4.25(1.66)
		1	11.4%	
		2	3%	
		3	6%	
		4	42.5%	
		5	13.8%	
		6	12.6%	
		7	10.8%	
	Post-test personal testimonies			4.89(1.76)
		1	3%	
		2	4.2%	
		3	7.2%	
		4	15.7%	
		5	24%	
		6	18%	
		7	18%	
	Post-test personal intuition			4.44(1.76)
		1	10.8%	
		2	4.2%	
		3	7.2%	
		4	28.1%	
		5	22.8%	
		6	12%	
		7	15%	
	Post-test traditionalism			4.82(1.78)
		1	7.2%	
		2	4.2%	
		3	8.4%	
		4	23.4%	
		5	16.2%	
		6	18%	
		7	22.8%	
	Post-test spirituality			4.60(1.55)
		1	6%	
		2	4.2%	
		3	7.8%	
		4	26.9%	
		5	26.9%	
		6	16.8%	
		7	11.4%	
Post-test attitudes toward egg cleanse				
	Post-test belief in effectiveness			5.02(1.52)
		1	3%	
		2	3.6%	
		3	9.6%	
		4	15%	
		5	28.7%	
		6	21.6%	
		7	18.6%	
	Post-test future use			4.81(1.77)
		1	7.8%	
		2	3.6%	
		3	10.2%	
		4	16.8%	
		5	21.6%	
		6	19.8%	
		7	20.4%	
	Post-test preference over medicine			4.12(2.03)
		1	18%	
		2	5.4%	
		3	13.8%	
		4	17.4%	
		5	18%	
		6	10.2%	
		7	17.4%	

Satisfaction with the physician				5.23(1.22)
	Agreement			5.08(1.41)
		1	9%	
		2	3.7%	
		3	4.6%	
		4	27.8%	
		5	24.1%	
		6	17.6%	
		7	21.3%	
	Satisfaction			5.35(1.36)
		1	1.9%	
		2	1.9%	
		3	4.6%	
		4	13%	
		5	31.5%	
		6	24.1%	
		7	23.1%	
	Competence			5.26(1.40)
		1	1.9%	
		2	3.7%	
		3	4.6%	
		4	13.9%	
		5	27.8%	
		6	28.7%	
		7	19.4%	
	Trust			5.02(1.54)
		1	4.6%	
		2	0.9%	
		3	10.2%	
		4	14.8%	
		5	30.6%	
		6	19.4%	
		7	19.4%	
	Openness			5.43(1.47)
		1	1.9%	
		2	0.9%	
		3	9.3%	
		4	13%	
		5	20.4%	
		6	25%	
		7	29.6%	

Note: All data in this table are descriptive (percentages, means, and standard deviations). No inferential tests were performed.

Table S4

Empathetic refutations and non-empathetic refutation.

Concept	Usage reason	Affirmation	Refutation
Holistic approach	Because it treats the person holistically, that is, in an integral way, considering their entire state of health and the body-mind relationship.	A positive lifestyle, good mental health and proper nutrition are important components in the fight against diseases. A comprehensive view of the person, which takes into account their personal context, should be of crucial importance when diagnosing and treating diseases. It is very good that you have these types of concerns and see health care as something that involves many parts of our lives.	Health professionals promote healthy lifestyles because they help prevent or treat some diseases, and we do so following ethical and scientific principles. It is important to differentiate between comprehensive health promotion and not paying adequate attention to a specific medical problem. Scientists and health professionals do not have evidence that egg cleanse promotes a healthy lifestyle. On the contrary, it is a practice that can cause problems such as not receiving a treatment that could work, or not receiving it in time, and the disease continuing to develop unnecessarily. It can also lead to confusion regarding the real origin of diseases, which in fact makes it difficult for people to develop healthy lifestyles that allow for good overall health. Egg cleanse is not part of medical treatments based on scientific evidence, developed through the most reliable knowledge available to us.
Natural treatments	Because I prefer natural treatments and products, free of chemicals or toxins.	Modern medicine would not be the same without natural remedies. For example, the use of willow bark as a painkiller dates back some 3,500 years. It was precisely this bark that formed the basis of some of the most widely prescribed painkillers today. It would be foolish to ignore natural remedies when developing treatments. Many of the effective treatments used today are based on nature in one way or another.	Part of the appeal of using egg cleanse is often that it would be more “natural,” even though there is no scientific reason to claim that using egg cleanse is more natural than using proven medical treatments. Furthermore, we cannot claim that something is better simply because it is more natural, because there are many toxic substances in nature, which would cause great harm or even death. The lack of side effects of egg cleanse is not because it is more natural, but because it has not been proven to actually have a real therapeutic effect on our body, so it cannot replace proven medical treatments. Scientifically based medical treatments must pass many safety checks. By valuing something only because it gives us the impression of being more natural, we could be missing the opportunity to receive a truly effective treatment for the disease we suffer from.

Dissatisfaction with conventional medicine	Because pharmaceutical companies and health authorities are corrupt and have economic interests.	In some cases, distrust of authorities and pharmaceutical companies is justified. Private companies in all sectors have an interest in making money with their products, so it is important to critically question their motivations. The attitude of some politicians can also be suspicious, especially when they politicize health programs. The critical attitude of citizens is of vital importance in a successful society. Critically questioning the motives of industries and politicians is very important, because lucrative activities sometimes involve a conflict of interest, cases of corruption, or can lead companies to issue misleading information.	Although we do not fully trust pharmaceutical companies and politicians, we have strong control mechanisms based on the best scientific methodologies. In Peru, the General Directorate of Medicines of the Ministry of Health carries out an intense activity of monitoring and approving medicines. The agencies that advise the government on the safety of medical treatments are made up of independent experts who are not under government control. Regulatory agencies take any potential safety issues very seriously. Unfortunately, these control mechanisms do not apply to products such as egg cleanse, even though the economic benefits are not limited to pharmaceutical companies. Sellers of egg cleanse also make a lot of money from it, even though it is a practice that is not properly controlled and can incur potential negative effects, such as not receiving a treatment that has been proven to be effective or not having a truly healthy lifestyle by confusing the real origin of diseases.
Benefits and security	Because it has greater benefits and is safer than conventional medical treatments.	It is normal to have questions and doubts about medical treatments and how they can affect us. We would all like medical treatments to be effective for everyone and in all conditions, but they cannot be guaranteed, like any other product, to be 100% safe and effective. Sometimes it is difficult to face uncertainty, so fear and rejection are perfectly understandable. Many people turn to alternative and traditional medicine without negative consequences, and many of these alternative remedies are presented as safer and more natural than conventional medical treatments.	The potential lack of side effects of egg cleanse is not because it is a better treatment, but because it has not been proven to actually have a real therapeutic effect on our body. Since a medical intervention, with a real effect on our body, can always have side effects, unlike egg cleanse, medical treatments undergo strict safety controls. Even though approved medical treatments are not 100% effective, they are approved because their benefits far outweigh any potential adverse effects. Moreover, if we wait to be absolutely certain of safety, we would never do anything in life. Imagine if we refused to get into a car unless the driver could prove 100% that we would not have an accident. Public health institutions and independent researchers have very reliable monitoring systems in place to track all possible side effects of drugs and other medical interventions, using statistics and taking into account many potential causes. However, we are often unaware of the effects and how responsibly egg cleanse is applied.
Personal testimonies	Because I trust the personal testimonies and experiences of people who have found it successful.	Health professionals and scientists must take into account the personal preferences and experiences of our patients, as testimonies are often useful in uncovering possible side effects that are rare or only occur in certain minority groups. Current medical practice is open to patients and their families actively participating in decision-making, which is important in order to ensure informed consent and achieve greater commitment to treatments. It is a good thing that you are attentive to the information you receive and that you are interested in your health.	Unlike scientifically supported medical treatments, techniques such as egg cleanse rely excessively on testimonies and personal experiences, which deviates from a more general view of the effectiveness and safety of medical treatments. On the one hand, testimonies are often unreliable because they are affected by many biases and misperceptions. For example, people may exaggerate or even lie if they are motivated by economic or ideological interests. On the other hand, the success of a medical treatment must be assessed by a cost/benefit calculation using large numbers of patients, sometimes millions, for which very reliable scientific methods have been developed. It is quite possible that by focusing only on the positive testimonials about egg cleanse we are overlooking negative experiences and their risks, such as not receiving a truly effective treatment in time or possible side effects.
Personal intuition	Because I trust my own intuition and my knowledge of my own body.	Doctors and scientists must discuss decisions with our patients taking into account their preferences and intuitions regarding the recommended treatments for them. We all, in one way or another, tend to listen to our intuition in certain situations where we must make important decisions. Sometimes it is even a good idea, since our intuition can lead us to make correct decisions that we would not reach if we thought too much about it. It is good to take into account your preferences and intuitive thoughts, since it can help us make an informed and consensual decision.	Medical experts must make decisions based on critical analysis of evidence. To have this reliable knowledge, we must critically evaluate the information and avoid misleading sources, as well as reduce our own prejudices and lack of knowledge. In contrast, techniques that are not supported by scientific and professional evidence, such as egg cleanse are based on data that is often distorted by personal biases and economic interests. For example, someone who advocates egg cleanse could consciously or unconsciously distort the data to suit his or her wishes, something that cannot happen in any way in medical science. Medicine is such a complex field of knowledge that even scientific researchers have to work in large groups, each member contributing their specific knowledge and skills. The information resulting from this collective analysis is therefore the most reliable source on which human knowledge can be based. It is sensible and intelligent to give more importance to these reliable sources when making decisions about something as important as our health.
Traditionalism	Because it is a traditional remedy that is part of my cultural identity.	Many of our traditions shape and give meaning to the way we act and identify ourselves, so they are part of who we are. People have the right to have their traditions taken into account by health professionals and to be treated with respect regardless of their cultural origin. In general, it is a positive thing to have appreciation for one's own culture, since it helps to preserve and develop it.	Practices that can be considered traditional, such as egg cleanse, do not have evidence of effectiveness when tested using rigorous scientific methods. Therefore, the use of egg cleanse, although it may seem old, can prevent us from receiving truly effective treatment, or deviate us from a healthy lifestyle by confusing the real origin of diseases. Having a lifestyle based on cultural traditions does not in any way imply endangering our lives and those of others, especially those who belong to risk groups or who have other traditions. Furthermore, just because egg cleanse is presented as something ancient or traditional does not mean that it is good for us or our societies to continue practicing it. Traditions are not sacred, unchangeable, immutable or good in themselves; they are constantly changing to suit our needs and ethical principles. Think of all the traditions that have changed throughout our lifetime in favor of healthier practices (for example, smoking in front of children or driving without a seat belt). We must keep ourselves alive if we want, in turn, to keep our traditions alive.
Spirituality	Because of my view of spirituality and the way energy works.	Health professionals must respect people's religious and spiritual beliefs, because it is understandable to be concerned about their compatibility with medical treatments. People want their decisions to be consistent with their deepest beliefs and values. However, it is common for scientists and doctors not to think in terms of spirituality. Sometimes it can even seem that there is a contradiction between certain forms of spirituality and medical science, for example, regarding the origin of diseases. All this can be confusing.	The main leaders of the most widespread religions in the world support and encourage the use of scientifically supported medicine, recognizing that there is no incompatibility with their beliefs, and this includes Christianity, Islam, Judaism, Buddhism and Hinduism. Problematic cases are very rare and are usually restricted to very minority forms of spirituality regarding a particular treatment. The protection of human life is always an act of love for other human beings and for oneself, and is consistent with moral, religious and spiritual values.

Non-empathetic	All	-	Although it is promoted as a spiritual practice, the use of egg cleanse has not been scientifically proven to be effective in treating diseases and can cause a number of risks, such as preventing us from receiving truly effective treatment. Egg cleanse can also confuse us about the true origin of diseases, which are usually caused by viruses or bacteria that invade us or by physical failures in the functioning of our body.<br>Alternative medicine techniques such as egg cleanse have not been proven effective for treating diseases and therefore should not be used as a treatment. A lot of effort has been put into studying these types of practices and their effectiveness has never been conclusively proven, using the best tools available for scientific research. Because of this, doctors should not recommend their use, instead recommending treatments that have been proven effective. In addition, people may confuse the real origin of their disease, which can lead to unhealthy lifestyles, as well as losing the opportunity to receive effective treatment to treat their disease. Another thing that can happen is that the person administering egg cleanse, who is usually not a registered and controlled doctor, commits some negligent practice that causes additional damage to the disease itself. Because of all this, I do not recommend using egg cleanse to treat diseases.<br>The use of alternative medicine techniques such as egg cleanse is not recommended by medical associations and scientifically endorsed treatment guides, since we do not have evidence to support its use. On the other hand, the potential dangers of techniques such as egg cleanse are well documented, since they can generate distorted ideas regarding the origin and treatment of diseases, as well as cause patients not to use treatments that are endorsed.<br>Medicine is a science that develops at great speed and that has very powerful tools to generate knowledge and develop treatments that work. Alternative medicine such as egg cleanse, on the other hand, does not have evidence and, consequently, its use for the treatment of diseases is not recommended from a medical point of view.

Table S5

Times each usage reason was preferred by group.

	Empathetic refutation	Non-empathetic refutation	Control
Holistic approach	11	7	9
Natural treatments	13	20	18
Dissatisfaction	6	5	2
Benefits and security	5	7	5
Personal testimonies	20	25	34
Personal intuition	8	8	9
Traditionalism	26	27	26
Spirituality	15	13	15

Table S6

Robustness checks: ANCOVA results controlling for pre-test scores.

Outcome variable	Covariate (PRE)	F (Group)	df	p-value	Partial η^2
Post-test of belief in the effectiveness of egg cleanse	Pre-test of belief in the effectiveness of egg cleanse	3.13	2, 163	$< .05$	.04
Post-test of belief in the effectiveness of other CAM techniques	Pre-test of belief in the effectiveness of other CAM techniques	5	2, 163	$< .01$	.06
Post-test of preference over conventional medicine	Pre-test of preference over conventional medicine	0.45	2, 163	$> .05$	.01
Post-test of usage reasons	Pre-test of usage reasons	4.01	2, 163	$< .01$	.05

Note: All models included experimental condition (tailored debunk, non-tailored debunk, and control) as a between-subjects factor and the corresponding pre-test score as a covariate. Normality of residuals and homoscedasticity were inspected and showed no substantial deviations from model assumptions. However, homogeneity of regression slopes was not met for future use,

for which the ANCOVA results are therefore not interpreted. These analyses were conducted as robustness checks and do not alter the main conclusions of the study.
